# Tanshinone IIA attenuates valvular interstitial cells’ calcification induced by oxidized low density lipoprotein via reducing endoplasmic reticulum stress

**DOI:** 10.1515/med-2023-0797

**Published:** 2023-09-25

**Authors:** Fang Chen, Dongqiang Yang, Yuhua Ru, Yu Bai, Xueliang Pei, Jie Sun, Shan Cao, Weiguang Wang, Aishe Gao

**Affiliations:** Department of Pathophysiology, Henan University of Traditional Chinese Medicine, Zhengzhou 450008, China; Department of Infectious Diseases, Henan Provincial Peoples’ Hospital, Zhengzhou 450003, China; Department of Medical Academy, Soochow University, Soochow 215021, China; Department of Cardiovascular Surgery, Henan Provincial Peoples’ Hospital, Zhengzhou 450003, China

**Keywords:** Tanshinone IIA, osteoblastic differentiation, ER stress, valvular interstitial cells

## Abstract

Recent studies revealed that endoplasmic reticulum (ER) stress played an emerging role of in valve calcification. Tanshinone IIA (TanIIA) has been a research hotspot in cardiovascular diseases. Previously we found that sodium TanIIA dampened the pathological phenotype transition of valvular interstitial cells (VICs) by affecting ER stress published in Chinese Journal. Here, we test the hypothesis that TanIIA attenuates the pro-osteogenic effects of oxidized low-density lipoprotein (oxLDL) in VICs by reducing induction of ER stress. Patients’ aortic valve (AV) was collected, and porcine VICs were cultured for *in vitro* model. ER stress markers were tested in human leaflets by immunostaining. Immunoblotting were used to test the osteoblastic factors such as Runx2, osteocalcin, and ER stress markers GRP78, CHOP, XBP1, etc. Alkakine phosphate (ALP) activity assay were used to test the activity of ALP kinase. Pro-inflammatory gene expression was detected by polymerase chain reaction. As a result, ER stress markers were elevated in patients’ calcified AVs. OxLDL induced osteogenesis and inflammation via promoting ER stress. TanIIA attenuated oxLDL induced ER stress. TanIIA also inhibited theosteoblastic factors and inflammatory cytokine expressions in VICs. In conclusion, our data provide evidence that TanIIA exerts anti-inflammation and anti-osteogenic effects in VICs by attenuating ER stress, and ER stress acts as an important regulator in oxLDL induced VICs’ phenotype transition.

## 1 Introduction

Calcified aortic valve diseases (CAVD) and aortic stenosis (AS) evolved from CVAD have been attributed to prolonged age-associated valvular degeneration for many years [1]. Until recently, the mechanisms of CAVD are still not well understood, but it is considered that CAVD is a disease which can be regulated actively. Valvular interstitial cells (VICs) are the most predominant component of heart valves, and play an important effect on the occurrence and development of CAVD. In the process of formation of CAVD, VIC differentiate into either a myofibroblast or an osteoblast [2], cause fibroplasia initially, followed by widespread calcification and inflammation infiltration of the aortic valve (AV) leaflets [3].

As known to all, atherogenic oxidized low-density lipoprotein (oxLDL) deposits in human AV lesions, especially in the part of calcified valves. Some *in vitro* research studies revealed that oxidized cholesterol stimulated the generation of calcified nodule by inducing VICs’ transformation [4] and oxLDL caused endoplasmic reticulum (ER) stress in VICs and vascular smooth muscle cells (VSMCs) [5].

The unfold protein response (UPR) which consists of three branches, protein kinase-like ER kinase (PERK), inositol-requiring protein 1α (IRE1α), and activation of transcription factor 6 (ATF6), could be activated by ER stress [6]. Among UPR’s complicated networks, C/EBP homologous protein (CHOP), dominant regulated by PERK and IRE1/XBP-1 signaling pathways, exerted an important effect on the process of ER stress-associated inflammation and cellular apoptosis [7]. When the expression of CHOP downregulated, the inflammation would be attenuated and the activation of caspase in cells exposed to LPS or diabetic stressors would be prevented [8]. In addition, some studies have indicated that ER stress played an emerging effect on the formation of valve calcification. PERK/ATF4/osteocalcin pathway and IRE1α/XBP1s/Runx2 pathway have been elucidated in oxLDL-induced VIC phenotype transition and nuclear factor-κB (NF-κB)-related inflammation. Thus, drugs to attenuate ER stress and related inflammatory response during pathogenic valve calcification are very meaningful [5].

Danshen, scientific name called *Salvia miltiorrhiza* Bunge, is a kind of traditional Chinese medicine material [9]. Tanshinone IIA (TanIIA), which is a primary bioactive constituent of Danshen, has been a research hotspot in cardiovascular diseases in the past few decades. Lots of biological activities of TanIIA such as vasodilation, anti-inflammation, antioxidant, anti-ischemia, antiarrhythmia, inhibition of hyperplasia, antiatherosclerosis and so on have been reported [9]. The usage of TanIIA and its derivative water-soluble sodium TanIIA as prescription for a series of cardiovascular diseases including inoperable AS were published in many Chinese Journals [10,11]. However, the investigations of TanIIA in CAVD mainly focus on its function of vasodilation or antihyperplasia, the effects of TanIIA on VICs are never explored.

We found that sodium TanIIA dampened the pathological phenotype transition of VICs by affecting ER stress published in Chinese Journal [12]. Here, we test the hypothesis that TanIIA weakens the pro-osteogenic effects of oxLDL on VICs by reducing induction of ER stress and inflammation.

## 2 Materials and methods

### 2.1 Sample collection

This study was approved by Ethic Committees of Henan Provincial Peoples’ Hospital and Henan University of TCM, strictly complied with the Declaration of Helsinki. All patients and controls have signed informed consent for sample collection and its use in this study. Calcified AV leaflets were obtained from 11 patients who had AV replacement on account of severe AS intraoperatively at Henan provincial peoples’ Hospital. “Thin” AV leaflets were obtained from six patients who had Bentall surgery because of acute aortic dissection or aneurysm as control group. All valves used in this study were tricuspid valves.

### 2.2 Isolation and culture of VICs

Porcine VICs were extracted from AV leaflets of pigs by the method of collagenase II (Invitrogen, Carlsbad, CA, USA, Cat. 17101015) digestion described previously [13]. Porcine valve leaflets were added to essential medium containing 1 mg/mL collagenase II at 37℃ for 30 min for digestion. Then endothelial cells were removed by vortex carefully. Fresh medium with same ingredient was added for digesting again for 4–6 h at same temperature. Vortex and repeated aspirating were performed for sufficient tissue decomposition, and the final suspension was spun at 1,000 rpm for 10 min to precipitate the cells. Cells were re-suspended and cultured in Dulbecco’s modified eagle’s medium (TransGen, Beijing, China, Cat. FI101-01), replenished with 100 U/mL penicillin, 100 µg/mL streptomycin, and 10% fetal bovine serum (Gibco, Carlsbad, CA, USA, Cat. 10091) in an incubator [14]. Cells of passages 3–6 were used for the next experiments when they reached 70–90% confluence. If needed, pharmacological reagents, like TanIIA (Sigma-Aldrich, Cat. 568-72-9) and tauroursodeoxycholic acid (TUDCA; Sigma-Aldrich, Cat. 35807-85-3) were added 1 h ahead of the addition of oxLDL, and Tan IIA were lysed in dimethyl sulfoxide (Sigma-Aldrich, Cat.67-68-5) first and then adjust to the final concentration extemporaneously.

### 2.3 Isolation of LDL and preparation of oxLDL

Human plasma LDL was purified by sequential ultracentrifugation (1.019–1.063 g/mL) and oxidized by 5 μM CuSO_4_ at 37℃ for 20 h. Excess EDTA-Na_2_ was used to terminate oxidation. The purity and electrical charge of oxLDL were examined by electrophoretic migration assay in agarose gels [14]. Oxidic degree was quantified by the content of thiobarbituric acid reactive substances [14]. All the above chemical reagents were bought from HuaWei chemical Company (Zhengzhou, China). Lipoproteins were used within 2 weeks after experimental preparation.

### 2.4 Immunohistochemistry

Immunostaining was performed to identify GRP78 and CHOP expression using mouse monoclonal antibodies from Abcam (Cambridge, USA, Cat. ab212054, ab11419). Briefly, 5 μm sections were de-paraffinized by washing with xylene and graded ethanol, then the heat retrieval of sections were performed with sodium citrate buffer. After blocking in 10% goat serum (Boster, Wuhan, China, Cat. AR0009), these antibodies were put into use: GRP78 (1:100 dilution) and CHOP (1:100), overnight at 4℃. Then a two-step horseradish peroxidase (HRP)-conjugated antimouse kit (Boster, Cat. BA1050) was used. Finally, staining was carried through reaction with diaminobenzidine (Boster, Cat.AR1022) and counterstaining with hematoxylin (Sigma-Aldrich, Cat 517-28-2). Image-Pro Plus (Media Cybernetics) was used for quantitative analysis of two sections of each AV leaflet.

### 2.5 Immunoblotting

Western blotting was applied to analyze Runx2, osteocalcin, GRP78, CHOP, XBP1s, β-actin, phosphorylated and total IRE1α, PERK. If not mentioned, the antibodies were bought from Santa Cruz Biotechnology, Inc. (Santa Cruz, CA) except GRP78, CHOP, and XBP1s (Abcam; Cat. ab212054, ab11419, ab241571). Porcine VICs were dissolved in commercial radio immunoprecipitation assay lysis buffer (Beyotime, Jiangsu, China, Cat.P0013B) in accordance with the manufacturer’s instructions. The protein extracted from cytoplasm was then broke down on 4–20% SDS-PAGE gels and moved to polyvinylidene difluoride membrane [5]. After blocking with 5% (wt/vol) skim milk for 1 h at indoor temperature, membranes were incubated with primary antibodies at 4℃ overnight, followed by incubation with the appropriate HRP-conjugated secondary antibody for 1 h [13]. Membranes were exploited with enhanced chemiluminescence system; β-actin was analyzed for normalization of protein loading in cytosol. In phosphorylation assay, general levels of IRE1α and PERK were used for normalization. Band density was analyzed with Quantity One Software (Bio-Rad, Hercules, CA).

### 2.6 Alkaline phosphatase (ALP) activity assay

In the calcification experiments, VICs were treated with oxLDL for 72 h in a mixture of 10 mM β-glycerophosphate (Sigma-Aldrich, St Louis, MO, USA, Cat. 154804-51-0), 100 nM dexamethasone (National Institute for the Control of Pharmaceutical and Biological Products, Beijing, China, Cat. 101116-201803), and 50 μg/mL ascorbic acid (Sigma-Aldrich, Cat. 50-81-7) [14]. After washing the cells three times with PBS, the cellular proteins were dissolved with 1% Triton X-100 (Sigma-Aldrich, Cat. 9036-19-5) in 0.9% NaCl medium and then centrifuged [15]. The supernatant solution was used for ALP activity assay by commercial kit (Jiancheng, Nanjing, China, Cat. A059-2-2).

### 2.7 Real-time quantitative reverse transcription PCR (qRT-PCR)

qRT-PCR was applied to detect the amount of mRNAs in VICs encoding interleukin-6 (IL-6), IL-8, and monocyte chemoattractant protein 1 (MCP-1). RNA was extracted from VICs by TRIZOL (TransGen, Cat.ET-101) and reverse transcripted into cDNA using the methods described formerly [13]. RT-PCR were conducted on a StepOnePlus^TM^ R-T PCR System (Applied Biosystems, Foster City, CA) using SYBR Premix Ex Taq^TM^ (Takara, Otsu, Japan, Cat. ARR037A). The whole of primers for quantitative analysis in this study are as below: IL-6 (Forward: 5′-ATC AGG AGA CCT GCT TGA TG-3′, Reverse: 5′-TGG TGG CTT TGT CTG GAT TC-3′) [15], IL-8 (Forward: 5′-ATG ACT TCC AAG CTG GCC GTG GCT-3′, Reverse: 5′-TCT CAG CCC TCT TCA AAA ACT TCT C-3′) [15], MCP-1 (Forward: 5′-GTC ACC AGC AGC AAG TGT C-3′, Reverse: 5′-CCA GGT GGC TTA TGG AGT C-3′), and β-actin (Forward: 5′-GAC CTG ACC GAC TAC CTC-3′, Reverse: 5′-GCT TCT CCT TGA TGT CCC-3′) [15]. All results were analyzed by 2^−∆∆Ct^ method.

### 2.8 Statistical analysis

The represented graphic data are rendered as mean ± standard deviation (SD). Using SPSS, version 13.0 (SPSS, Chicago, USA) for statistical analysis. Statistical differences between the two groups were compared by student’s *t*-test, and statistical differences in multiple groups were calculated via one-way analysis of variance after confirming the normality of all the variables using the Kolmogorov–Smirnov test [14]. *P* < 0.05 was deemed to be significant statistically.

## 3 Results

### 3.1 ER stress were involved in patients’ calcified AVs

**Figure 1:**
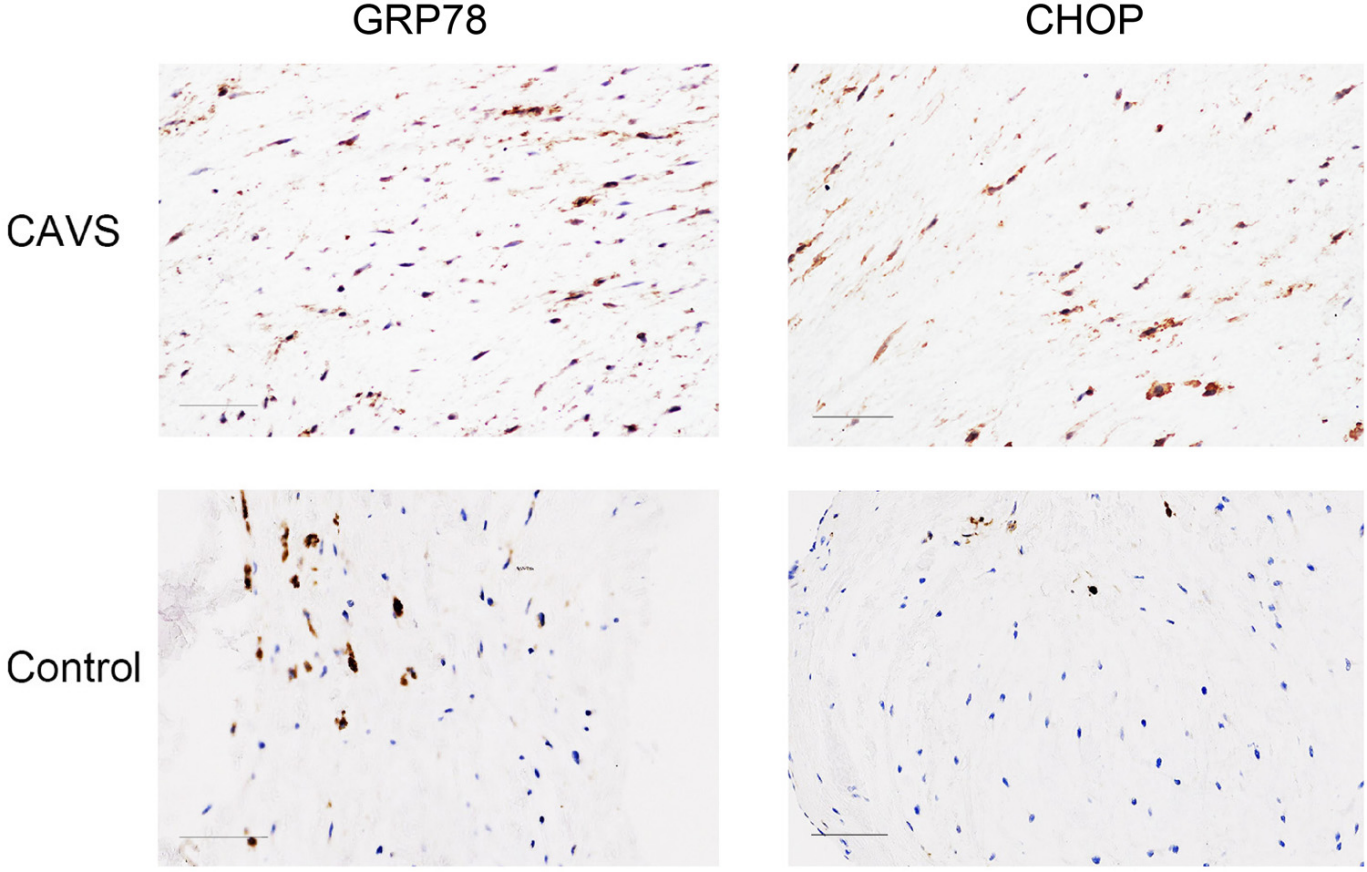
Representative immunochemical staining using GRP78 and CHOP antibody of representative chronic aortic valve stenosis (CAVS) patients and the control. The GRP78 and CHOP expression were increased in CAVS patients, scale bar = 100 μm.

**Figure 2:**
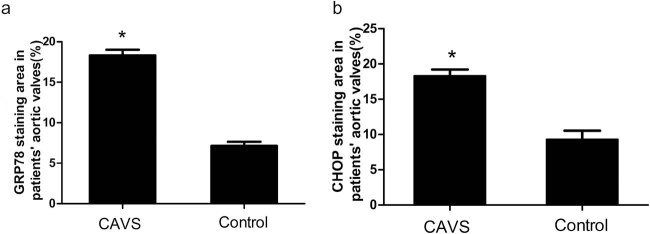
Quantitative analysis of the immunochemical staining (data are rendered as mean ± SD). (a) Indicates GRP78 staining area and (b) indicates CHOP staining area. *P < 0.05 vs control; *n* = 11 of CAVS patients, *n* = 6 of the control.

Previous literature demonstrated that ER stress was observed in human calcific valves. We also found that ER stress existed in degenerative calcified aortic leaflet by immunostaining, GRP78 and CHOP expressed highly in patients’ aortic leaflet (Figure 1) and quantitative analysis confirmed the results (Figure 2). For all these patients, their clinical data were collected and are listed in Table 1.

**Table 1:** Clinical characteristics of patients with calcified AS and the controls

	Patients	Controls	*P* value
Numbers	11	6	
Age (years)	58.3 ± 4.2	60.1 ± 2.8	n.s.
Sex (male)	6(54.5)	3(50)	n.s.
Body mass index	23.2 ± 1.8	23.6 ± 1.4	n.s.
**Risk factors**			
Hypertension	7(63.6)	4(66.7)	n.s.
Hypercholesterolemia	5(45.5)	3(50)	n.s.
Diabetes mellitus	3(27.3)	2(33.3)	n.s.
Smoking	4(36.7)	2(33.3)	n.s.
**Medications**			
Statins	9(81.8)	5(83.3)	n.s.
ACEI/ARB	5(45.5)	3(50)	n.s.
β-Blockers	5(45.5)	3(50)	n.s.
**Echocardiographic parameters**			
LVEF (%)	55.6 ± 5.8	50.2 ± 6.7	<0.05
Transvalvular gradient (mmHg)	80.5 ± 5.5	15.8 ± 3.5	<0.05
AV area (cm^2^)	0.6 ± 0.1	3.1 ± 0.4	<0.05
Aortic insufficiency	9(81.2)	5(83.3)	n.s.

Notes: All the values are presented as mean ± SD or *n* (%). ACEI: angiotensin-converting enzyme inhibitor; ARB: angiotensin receptor blocker; LVEF = left ventricular ejection fraction; n.s.: not significant.

### 3.2 TanIIA attenuated oxLDL induced osteoblastic differentiation and pro-inflammatory genes expression

**Figure 3:**
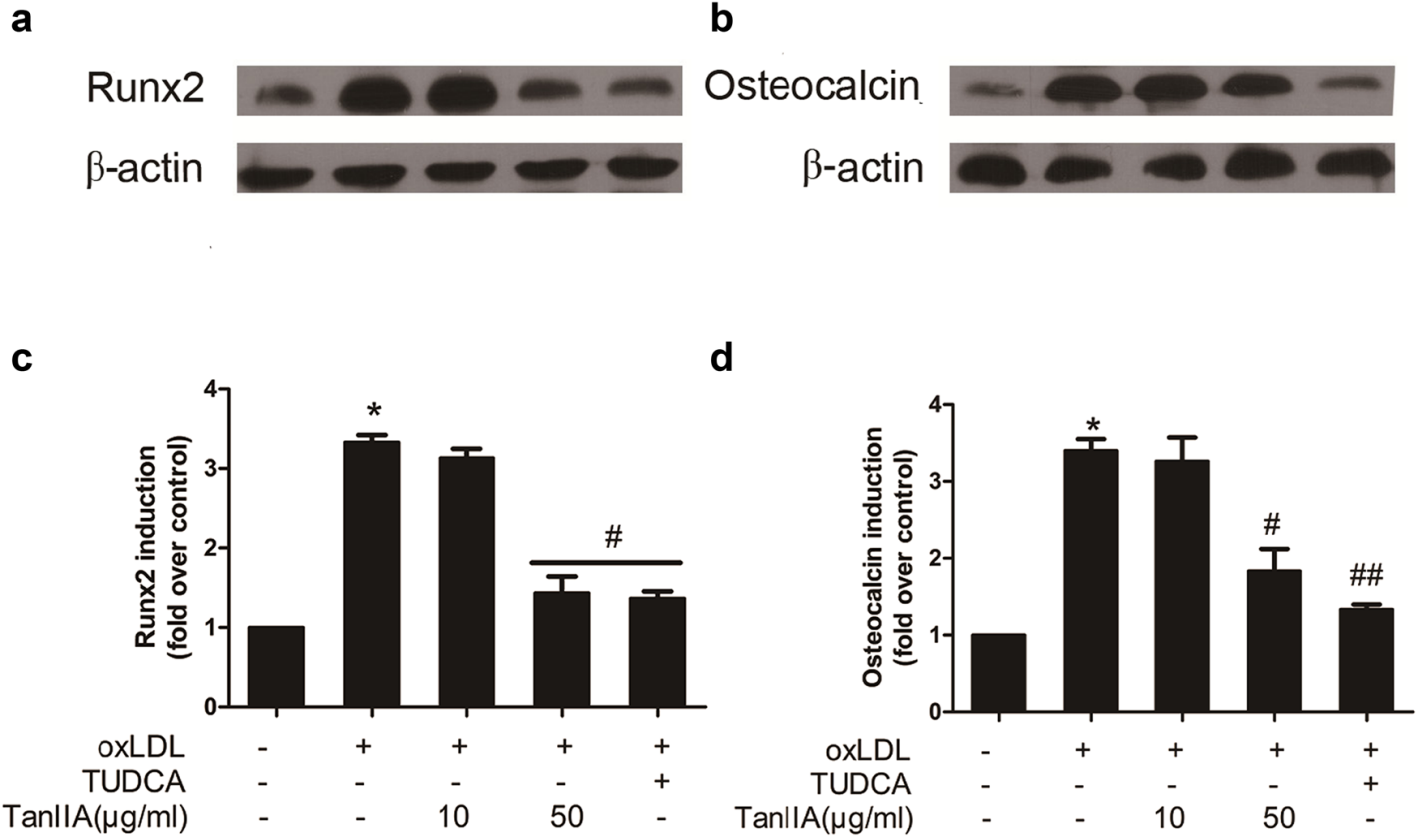
TanIIA attenuated oxLDL induced pro-osteogenic factor expressions in VICs. OxLDL induced Runx2 (a) and osteocalcin (b) expression in VICs, TanIIA decreased Runx2 (c) expression and osteocalcin (d) at 50 μg/mL. (Data are rendered as mean ± SD of independent experiments in cells from three different samples. *P < 0.05 vs control; ^#^ *P* < 0.05 vs oxLDL; ^##^ *P* < 0.01 vs oxLDL; TUDCA: tauroursodeoxycholic acid; TanIIA: Tanshinone IIA).

OxLDL is proved to initiate VICs phenotype transition *in vivo*. We treated the cultured VICs with or without oxLDL (100 μg/mL) for 72 h. TanIIA (10, 50 μg/mL) or TUDCA (1 μmol/L), a classic ER stress inhibitor, was used as intervention to the cells 1 h prior to incubation with oxLDL. Then we examined the expression of Runx2 gene (Figure 3a) and osteocalcin (Figure 3b) in different treatment groups by immunoblot, and the quantitative analysis was performed (Figure 3c and d). Runx2, also designated Cbfa1, is a master transcription factor for bone formation, and osteocalcin is an osteoblast-related gene that enhances mineralization [16]. OxLDL upregulated the expression of Runx2 gene and osteocalcin, and the effects were diminished by TanIIA (50 μg/mL) and TUDCA.

Accompanying with the promoted expression of pro-osteogenic factors, the pro-inflammatory cytokines like IL-6, IL-8, and MCP-1 (Table 2) were also elevated by oxLDL, the effects were also diminished by higher dose of TanIIA (50 μg/mL) and TUDCA.

**Table 2:** TanIIA attenuated oxLDL induced pro-inflammatory cytokines releasing (*n* = 3)

Group	mRNA expression (fold over control)		
	IL-6	IL-8	MCP-1
Control	1	1	1
oxLDL	3.75 ± 0.25^a^	4.33 ± 0.30^a^	3.68 ± 0.15^a^
TanIIA + oxLDL	2.20 ± 0.15^b^	3.12 ± 0.08^b^	2.05 ± 0.08^b^
TUDCA + oxLDL	2.20 ± 0.21^b^	2.42 ± 0.11^b^	2.07 ± 0.10^b^

^a^ *P* < 0.05 vs control; ^b^ *P* < 0.05 vs oxLDL; TUDCA: tauroursodeoxycholic acid; TanIIA: Tanshinone IIA.

### 3.3 TanIIA inhibited calcification in cultured VICs

The calcification of cultured VICs was measured by ALP activity in calcified medium mentioned above for required time interval. TanIIA and TUDCA were added 1 h before oxLDL. OxLDL significantly facilitated the ALP activity (Table 3), while TanIIA counteracted the effects.

**Table 3:** TanIIA decreased oxLDL-induced ALP activity of VICs (*n* = 3)

Group	ALP activity (normalized)
Control	1
oxLDL	4.22 ± 0.25^a^
TanIIA (10 mg/mL) + oxLDL	3.85 ± 0.30
TanIIA (50 mg/mL) + oxLDL	2.80 ± 0.21^b^
TUDCA + oxLDL	2.00 ± 0.16^b^

^a^ *P* < 0.05 vs control; ^b^ *P* < 0.05 vs oxLDL; TUDCA: tauroursodeoxycholic acid; TanIIA: Tanshinone IIA.

### 3.4 TanIIA attenuated ER stress and ER stress related nuclear factor expression

**Figure 4:**
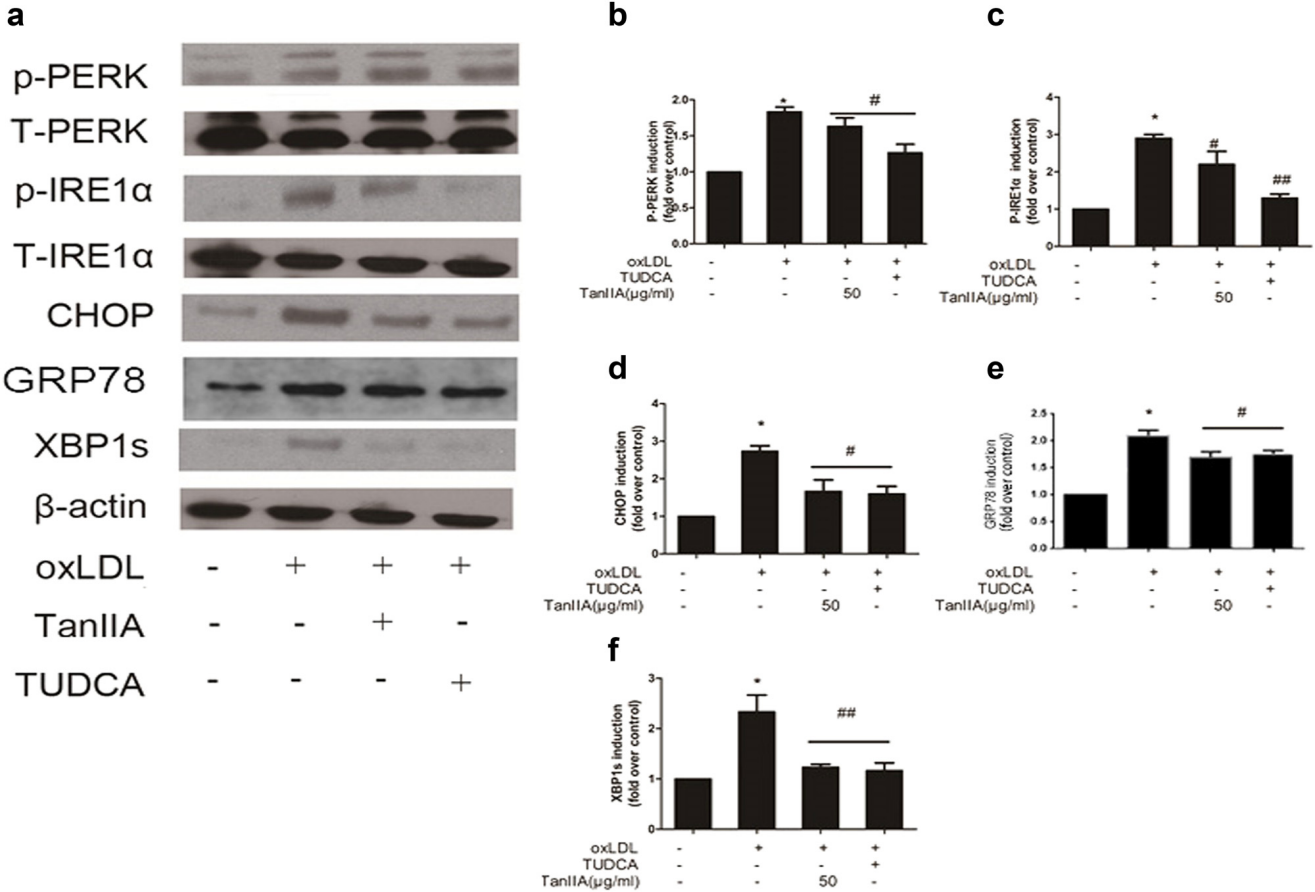
OxLDL augmented ER stress in VICs and TanIIA reduced the effects. The augmented expression of p-PERK, p-IRE1α, GRP78, CHOP, and XBP1 were reduced by TanIIA (a) and quantitative analysis confirmed the results (b–f). (Data are presented as mean ± SD of independent experiments in cells from three different samples. *P < 0.05 vs control; ^#^ *P* < 0.05 vs oxLDL; TUDCA: tauroursodeoxycholic acid; TanIIA: Tanshinone IIA).

The ER stress induction of UPR consists of three branches, only IRE1α and PERK pathways were demonstrated with valve calcification. We tested the two pathways using immunoblot, and found that the elevation of p-PERK, p-IRE1α CHOP, and XBP1 in cells dealt with oxLDL. Additionally, we also found that TanIIA inhibited the oxLDL-induced ER stress markers similar to ER stress inhibitor TUDCA (Figure 4a). The results were further confirmed by quantitative analysis (Figure 4b–f).

## 4 Discussion

In this study, the molecular mechanisms of TanIIA in the occurrence and development of CAVD were explored for the first time. We found that TanIIA exerted anti-inflammation and anti-osteogenesis effects in VICs by attenuating ER stress and related inflammation.

The UPR, which is capable to sense the protein misfolding and transmit this message to gene expression programs in ER, plays important roles in establishing and maintaining cellular homeostasis, particularly in cells secreting highly, such as VICs that engender a lot of key factors for osteogenesis and osteoblasts [5,17–19]. Usually, the UPR activates genes like ATF6, IRE1, and PERK, and also regulates the expression of some target genes, such as CHOP, BIP, and XBP1 [18]. The deletion of UPR signaling proteins such as ATF4 results in abnormal bone development and loss of bone phenotype in animal model [20]. Cai et al. also demonstrated that osteoblastic differentiation and inflammatory responses induced by oxLDL in VICs could be inhibited via restraining the ER stress using either tauroursodeoxycholic acid or 4-phenyl butyric acid [5]. On the contrary, prolonged and unmitigated UPR switches to initiate cell apoptosis program and intervertebral disc degeneration [21], which is largely mediated by the GRP78 and (CHOP)-GADD34 signaling axis [18,21]. Similar to Cai’s work, we proved that ER inhibition would attenuate osteogenesis and inflammation in VICs. Although the detailed mechanism in ER stress related osteogenesis needs to be investigated deeply, it is tempting to speculate that ER inhibitor could be useful in the future to deal with osteogenesis.

TanIIA has been proved to be a selective ER suppressor in certain cell types except tumor cells [22], and it exerts ability to affect transcription factors such as NF-κB [23–25]. TanIIA regulated TNF-α-induced expression of cytokines such as VCAM-1 and ICAM-1 via restraining NF-κB’s activation [26], inhibited pro-inflammatory cytokines such as IL-1, IL-6, TNF-α, and MCP-1 expression induced by oxLDL [27,28], which supported our *in vitro* studies.

Mineralization and VICs phenotype transition are characteristics of AV lesions, and arise in the area close to inflammation [29]. To this point, the anti-inflammation function of TanIIA is very commendable. We found that ER stress dependent pathways, especially for PERK/ATF4/osteocalcin pathway and IRE1α/XBP1s/Runx2 pathway, mediated anti-inflammatory effects of TanIIA in VICs; meanwhile, these pathways might also be the key point of osteogenesis.

## 5 Conclusions

As far as we know, the molecular mechanisms of TanIIA in valve calcification are illuminated for the first time and the relationship between ER stress and CAVD are further improved in this study. In China, a lot of traditional Chinese doctors like to use Danshen herbs and these findings will give strong evidence to the clinical use of TanIIA and related herbs in the treatment of CAVD patients from an experimental level. Taken together, we found that TanIIA perturbed the process of inflammation and osteogenesis in VICs *in vitro* through the PERK/ATF4/osteocalcin pathway and IRE1α/XBP1s/Runx2 pathway. Although more evidence *in vivo* is needed, the present study suggests that TanIIA might be a potential option for the treatment of CAVD.
